# The PI3Kδ inhibitor roginolisib (IOA‐244) preserves T‐cell function and activity

**DOI:** 10.1002/1878-0261.70203

**Published:** 2026-01-22

**Authors:** Elise Solli, Alessio Bevilacqua, Mathias Wenes, Denis Migliorini, Lars van der Veen, Sigrid S Skånland, Giusy Di Conza, Kjetil Taskén

**Affiliations:** ^1^ Department of Cancer Immunology Institute for Cancer Research, Oslo University Hospital Norway; ^2^ KG Jebsen Centre for B‐cell Malignancies, Institute of Clinical Medicine, University of Oslo Norway; ^3^ iOnctura SA Geneva Switzerland; ^4^ Faculty of Medicine, University of Geneva Switzerland; ^5^ Center for Translational Research in Onco‐Hematology, University of Geneva Switzerland; ^6^ Department of Oncology Geneva University Hospitals (HUG) Switzerland; ^7^ iOnctura BV Amsterdam the Netherlands

**Keywords:** CD4^+^ T helper cell, CD8^+^ cytotoxicity, chronic lymphocytic leukemia, PI3K inhibitor, regulatory T cells, T‐cell function

## Abstract

PI3K inhibitors (PI3Ki) have shown promise in some hematological cancers, but further development has been hampered by reports of serious immune‐related adverse effects. Thus, identification of effective PI3Ki lacking these adverse effects is desirable. Here, we evaluated the *in vitro* effects of the investigational PI3Ki roginolisib (IOA‐244) and the approved PI3Ki idelalisib on immune cells and leukemic cells. Roginolisib inhibited chronic lymphocytic leukemia cell signaling and viability in a manner comparable to idelalisib. Both drugs specifically inhibited PI3K‐signaling in T cells, validating their on‐target effects. Both idelalisib and roginolisib reduced regulatory T‐cell frequency in a concentration‐dependent manner, with idelalisib demonstrating greater potency. Both inhibitors also reduced T‐cell activation and proliferation, but to differing extents. However, only idelalisib induced a pronounced impairment of CD8^+^ T‐cell cytotoxic function. Furthermore, idelalisib treatment promoted differentiation of conventional CD4^+^ T cells into Th1, Th2, and Th17 subsets—a response not observed with roginolisib. In summary, roginolisib functions as an effective PI3K inhibitor on leukemic cells while preserving T‐cell functions, posing it as an alternative to current PI3K inhibitors.

## Graphical abstract

Identification of novel PI3K inhibitors with limited immune‐related adverse effects is highly sought after. We found that roginolisib and idelalisib inhibit chronic lymphocytic leukemia (CLL) cells and Treg suppressive functions to similar extents, but roginolisib affects cytotoxic T‐cell function and promotion of pro‐inflammatory T helper subsets to a much lesser extent than idelalisib.

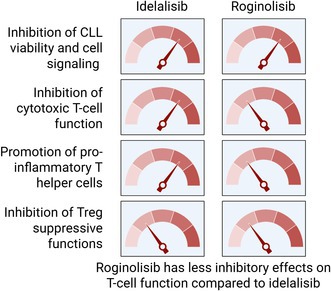

**Abbreviations**
CLL: chronic lymphocytic leukemia
PBMCs: peripheral blood mononuclear cells
PI3K: phosphatidylinositol 3‐kinase
PI3Ki: PI3K inhibitors
TCR: T‐cell receptor
Teffs: T effector cells
Tregs: regulatory T cells

## 1 Introduction

Dysregulation of the phosphoinositide 3‐kinase (PI3K) pathway is observed in a large number of cancers, and its hyperactivation is believed to be a significant malignant driver [1, 2]. Thus, there has been great interest in developing drugs that can target and inhibit this pathway. The PI3K proteins can be divided into three classes based on their structure and substrate specificity, namely class I, II, and III, where class I is the most extensively studied [3, 4]. The class I PI3K proteins consist of a regulatory subunit (p85) and a catalytic subunit (p110), where the catalytic subunit can be one of four isoforms (p110α, p110β, p110γ, or p110δ). PI3K transmits a cellular signal by converting PI(4,5)P2 to PI(3,4)P3 using ATP, and PI(3,4,5)P3 further regulates downstream targets, such as PDK1, AKTt, and mTOR [5]. This signaling pathway then regulates key cellular functions, including cell survival, proliferation, metabolism, and migration.

Idelalisib was the first class I PI3Ki that received FDA approval in 2014 for the treatment of certain B‐cell malignancies [3]. In the following years, other PI3Ki were in development, either as a monotherapy or in combination with other drugs, namely, the pan‐class I PI3K inhibitor copanlisib, the dual PI3Kδ/casein kinase 1ε inhibitor umbralisib, the dual PI3Kδ/γ inhibitor duvelisib, and the PI3Kα inhibitor alpelisib. These PI3K exert their functions by blocking ATP‐binding to the catalytic domain, thereby disabling the conversion of PI(4,5)P2 to PI(3,4,5)P3 and inhibiting the further transmission of the cellular signal. Even though these drugs have shown promise, their further development has been challenging due to safety concerns, including reports of immune‐related adverse effects [6, 7]. Following these concerns, several of the PI3K inhibitors have been withdrawn from the market or issued with black box warnings. Thus, the development of novel, effective PI3K without these toxic adverse effects would be highly desirable.

Here, we evaluated the effects of the novel non‐ATP competitive PI3Kδ inhibitor roginolisib (IOA‐244) compared with the approved PI3K inhibitor idelalisib. Roginolisib has been shown to be well tolerated, has a manageable toxicity profile [8], and is currently in clinical trials for myelofibrosis (NCT6887803), uveal melanoma (NCT06717126), refractory/relapsed chronic lymphocytic leukemia (CLL) (NCT06644183), nonsmall cell lung cancer (NCT06879717), and other metastatic cancers (NCT04328844). We found that roginolisib inhibits CLL B‐cell receptor signaling and viability similarly to idelalisib. Both drugs also inhibited PI3K‐mediated signaling specifically in T cells in a concentration‐dependent and comparable manner, showcasing the specificity of these drugs. However, multiple *in vitro* assays using adaptive immune cells from healthy peripheral blood mononuclear cells (PBMCs) revealed distinct differentiation profiles between roginolisib and idelalisib, which cannot be fully attributed to differences in potency alone. These findings suggest that roginolisib may better preserve T‐cell function compared with idelalisib in a cancer context. Consequently, further investigation is warranted to determine whether this translates into a more favorable adverse effect profile.

## 2 Materials and methods

### 2.1 Reagents and antibodies

Idelalisib was obtained from MedChemExpress (Monmouth Junction, NJ, USA) and roginolisib was obtained from iOnctura (Geneve, Switzerland).

Antibodies for flow cytometry staining that were purchased from BD BioSciences (Franklin Lakes, NJ, USA) include anti‐FoxP3‐PE‐CF594 (clone: 259D/C7), anti‐CD3‐APC‐H7 (clone: SK7), anti‐CD19‐PE (clone: HIB19), anti‐CD8‐PE (clone: RPA‐T8), anti‐CD8‐PE‐Cy7 (clone: RPA‐T8), anti‐CD25‐PE (clone: M‐A251), anti‐CD127‐Alexa647 (clone: HIL‐7R‐M21), and anti‐CTLA‐4‐APC (clone: BNI3). Anti‐PD‐1‐BV421 (clone: EH12.2H7), anti‐CD4‐FITC (clone: SK3), CD3‐FITC (clone: OKT3), CD4‐APC‐Cy7 (clone: SK3), CD25‐BV605 (clone: BC96), CD45RA‐BUV395 (clone: HI100), CD127‐BV711 (clone: A019D5), CXCR3‐BV421 (clone: G025H7), CCR4‐PE‐Cy7 (clone: L291H4), CCR6‐PE (clone: G034E3) and CCR10‐APC (clone: 6588_5) were purchased from BioLegend (San Diego, CA, USA); anti‐TCF‐1‐Ax488 (clone: C63D9) was purchased from Cell Signaling Technologies (Danvers, MA, USA), and anti‐TOX‐PE (clone: TXRX10) and CXCR5‐PerCP‐eF710 (clone: SPRCL5) was purchased from Invitrogen (Waltham, MA, USA). Phosphospecific antibodies purchased from BD BioSciences include anti‐CD3ζ (pY142, clone: K25‐407.69), anti‐Zap70/Syk (pY319/Y352, clone: 17A/P‐ZAP70), anti‐SLP‐76 (pY128, clone: J141‐668.36.58), and anti‐mTOR (pS2448, clone: O21‐404). Anti‐PI3K p85/p55 (pY458/Y199, clone: E3U1H), anti‐Akt (pY273, clone: D9E), anti‐Akt (pT308, clone: D25E6), anti‐cleaved caspase‐3 (clone: D3E9), and anti‐S6 ribosomal protein (pS235/236, clone: D57.2.2E) were purchased from Cell Signaling Technologies. All phosphospecific antibodies were conjugated to Alexa 647, and all antibodies were raised against human variants of the protein.

### 2.2 Isolation of healthy donor T cells

Healthy donor buffy coats were either obtained from anonymized and informed blood donors from Oslo University Hospital Blood Centre (Oslo, Norway) according to Regional Ethics Committee approval (REK: 280751), or from de‐identified healthy human volunteers obtained from the Center of Interregional Blood Transfusion SRK Bern between April 2024 and November 2025. All donors provided written consent, and the study was conducted in accordance with the Declaration of Helsinki. T cells were isolated using the RosetteSep Human T Cell Enrichment Cocktail (STEMCELL Technologies, Vancouver, Canada) followed by gradient centrifugation using LymphoPrep (STEMCELL Technologies) according to the manufacturer's instructions. Isolated cells were cultured in Roswell Park Memorial Institute (RPMI) media with L‐glutamine (VWR, Radnor, PA, USA) supplemented with 10% fetal bovine serum (FBS) from Gibco (Billings, MT, USA), 1% sodium pyruvate (Gibco), 1% nonessential amino acids (Gibco), and 1% penicillin–streptomycin (Sigma Aldrich, St. Louis, MO, USA) at 37 °C and 5% CO_2_. This medium is referred to as complete medium.

### 2.3 Isolation of CLL PBMCs

CLL patient blood samples were obtained from the Department of Haematology, Oslo University Hospital, Norway from informed patients who provided written consent from August 2016 to November 2022. Patient characteristics are listed in Table S1. The study was approved by the Regional Ethics Committee (REK: 2016/947) and in accordance with the Declaration of Helsinki. Isolation of CLL PBMCs was done by gradient centrifugation using LymphoPrep (STEMCELL Technologies) according to the manufacturer's instructions. The PBMCs were cryopreserved, which does not affect the functionality of the cells [9].

### 2.4 CLL viability assay

CLL PBMCs were co‐cultured with fibroblast feeder cells to prevent spontaneous apoptosis of the CLL cells and mimic the tumor microenvironment, as described previously [10]. Briefly, a mixture of irradiated murine NIH/3 T3 fibroblasts stably expressing the ligands A proliferation‐inducing ligands (APRIL), B‐cell‐activating factor (BAFF), or CD40‐L (also co‐expressing APRIL) were co‐cultured with CLL PBMCs. The different fibroblasts were seeded at a 1 : 1 : 1 ratio, and the fibroblasts were seeded at a 1 : 10 ratio to the CLL PBMCs. The cells were co‐cultured in complete medium for 24 h at 37 °C and 5% CO_2_. Next, the CLL PBMCs were removed from the adherent fibroblasts, treated with 0.1% DMSO or indicated concentrations of idelalisib or roginolisib for 72 h in complete medium at 37 °C and 5% CO_2_. The cells were then exposed to CellTiterGlo reagents (Promega, Madison, WI, USA) to assess cell viability, according to the manufacturer's instructions. Luminescence was measured using an Envision plate reader (PerkinElmer, Waltman, MA, USA).

### 2.5 Flow cytometry

The cells were washed with PBS and stained with Fixable Viability Dye (Alexa Flour 700, Thermo Fischer Scientific, Waltham, MA, USA) for 20 min at room temperature (RT). Next, the cells were washed with PBS + 2% FBS and stained with fluorescently labeled antibodies against surface markers for 30 min at RT. The cells were then fixed using the Human FoxP3 Buffer Set Buffer A (BD BioSciences) for 10 min at RT before being permeabilized using the Human FoxP3 Buffer Set Buffer C (BD BioSciences) for 30 min at RT, according to the manufacturer's instructions. Lastly, the cells were stained with intracellular markers for 30 min at RT. The cells were resuspended in PBS + 2% FBS and analyzed on a BD LSR Fortessa or BD FACSymphony flow cytometer and analyzed using FlowJo (v10.8.1, Becton Dickinson & Company (BD)).

### 2.6 T‐cell characterization and proliferation assay

T cells were isolated as described above, stained with Cell Trace carboxyfluorescein succinimidyl ester (CellTrace Violet or CellTrace CFSE, Invitrogen) according to the manufacturer's instructions, and seeded at 3 × 10^5^ cells per well in 96‐U well bottom plates and treated with indicated concentrations of idelalisib and roginolisib (or duvelisib where indicated), and activated with anti‐CD3/2/28 beads (1 : 1 : 1 ratio, T Cell Activation/Expansion Kit, Miltenyi Biotec, Cologne, Germany) at 1 : 2 bead‐to‐cell ratio for 2–7 days at 37 °C and 5% CO_2_. For CD4^+^ T conv characterization, 2.5 × 10^5^ cells were activated in a 48‐well plate with TransAct (Miltenyi), 20 IU·mL^−1^ recombinant human IL‐2, and roginolisib, idelalisib, or DMSO control, and stimulated in 24‐well plates coated with anti‐CD3/28 (1 μg·mL^−1^ in PBS). Following drug treatment, the cells were stained with anti‐PD‐1‐BV421 and anti‐CTLA‐4‐APC as surface markers, and anti‐FoxP3‐PE‐CF594 and anti‐TOX‐PE as intracellular markers and subjected to flow cytometry analysis. For CD4 T conv characterization, cells were stained with CD3‐FITC, CD4‐APC‐Cy7, CD25‐BV605, CD45RA‐BUV395, CD127‐BV711, CXCR3‐BV421, CXCR5‐PerCP‐eF710, CCR4‐PE‐Cy7, CCR6‐PE, and CCR10‐APC. T‐cell proliferation was assessed by flow cytometry and indicated by a decrease in CFSE staining.

### 2.7 Treg suppression assay

Tregs, CD4^+^ T effector cells (Teffs), and CD8^+^ Teffs were isolated from healthy donor T cells using the CD4^+^CD25^+^CD127^dim/−^ Regulatory T Cell Isolation Kit II (Miltenyi Biotec), following the manufacturer's instructions. 5 × 10^4^ Tregs were seeded in 96‐well V‐bottom plates and treated with indicated concentrations of the drugs or DMSO and anti‐CD3/2/28 beads (T Cell Activation/Expansion Kit, Miltenyi Biotec) at 1 : 5 bead‐to‐cell ratio for 2 days at 37 °C and 5% CO_2_. The CD4^+^ and CD8^+^ Teffs were cultured at 5 million cells·mL^−1^ in complete medium in parallel. Following 2 days of incubation, the Tregs were stained with anti‐CTLA‐APC, anti‐PD‐1‐BV421, and anti‐TOX‐Alexa594 and subjected to flow cytometry analysis, or the donor‐matched Teffs were stained with Cell Trace carboxyfluorescein succinimidyl ester (CellTrace Violet or CellTrace CFSE, Invitrogen) according to the manufacturer's instructions and co‐cultured with the Tregs. The treated Tregs were washed of the drugs, and Tregs and Teffs were seeded at a 1 : 5 ratio (Teffs‐to‐Tregs) with anti‐CD3/2/28 beads at a 1 : 5 ratio (beads‐to‐cell) for 4 days. After 4 days incubation, the cells were prepared for flow cytometry as described above and stained with anti‐CD8‐PE before analysis on a BD LSR Fortessa flow cytometer.

### 2.8 Phosphoflow cytometry and fluorescent cell barcoding

Phosphoflow cytometry was conducted as previously described [11]. For phosphoflow in CD3^+^ T cells, the cells were treated with DMSO or indicated concentrations of idelalisib or roginolisib for 30 min at 37 °C. TCR signaling was induced by addition of biotinylated anti‐CD3 (5 μg·mL^−1^, Invitrogen), anti‐CD28 (5 μg·mL^−1^, Invitrogen), and anti‐CD2 (5 μg·mL^−1^, Invitrogen) for 2 min, followed by addition of avidin (50 μg·mL^−1^, Zymed, Seattle, WA, USA) to induce TCR signaling via cross‐linking. Next, the cells were fixed at designated timepoints in prewarmed BD Phosflow Fix Buffer I (BD Biosciences) for 10 min. For phosphoflow in CLL, PBMCs were co‐cultured with fibroblasts as described above, before being treated with DMSO or indicated concentrations of idelalisib or roginolisib for 24 h in complete medium for 24 h at 37 °C and 5% CO_2_. Next, B‐cell receptor signaling was initiated by addition of 10 μg·mL^−1^ anti‐IgM (Cell Signaling Technologies, Danvers, MA, USA). The cells were then fixed after 5 min in prewarmed BD Phosflow Fix Buffer I (BD Biosciences) for 10 min.

The fixed T cells or CLL PBMCs were washed and fluorescently barcoded as previously described [12] using unique combinations of Alexa Flour 488, Pacific Orange, and Pacific Blue (Thermo Fisher Scientific) at different concentrations in the presence of 0.02% saponin in PBS, for 20 min at RT. Cells were then pooled and permeabilized with the Human FoxP3 Buffer set buffer C for 30 min at RT to ensure nuclear permeabilization, followed by a second permeabilization with ice‐cold Phosflow Perm Buffer III (BD Biosciences). Cells were kept at −80 °C for at least 30 min or until further analysis. Finally, cells were washed and stained with phosphospecific‐ and cell surface antibodies for 30 min at RT and run on a BD LSR Fortessa flow cytometer (BD Biosciences). Data analysis was performed using Cytobank (https://cellmass.cytobank.org/cytobank/). The phosphorylation signal was calculated as the inverse hyperbolic sine (arcsinh) of the median fluorescent intensity (MFI) of the phosphorylation signal in unstimulated cells versus stimulated cells at indicated timepoints. The background phosphorylation signal obtained from isotype control was subtracted from the phosphorylation signal of each phosphoantibody.

### 2.9 Cytotoxicity assay

T cells were isolated as described above, before CD8^+^ T cells were enriched using CD8 Microbeads (Milentyi Biotec) according to manufacturer's instructions. The cells were treated with DMSO, idelalisib, roginolisib, or duvelisib at 5 μm and activated with anti‐CD3/2/28 beads (1 : 2 beads‐to‐cell ratio) for 7 days at 37 °C and 5% CO_2_. The cell culture medium was changed after 2 days of incubation and re‐supplemented with the compounds at indicated concentrations. After 7 days of treatment, the cells were washed before being co‐cultured with calcein‐stained mouse mastocytoma cell line (P815) expressing mouse Fc receptor. Calcein staining was conducted according to the manufacturer's instructions (Abcam, Cambridge, UK), and calcein release was used as a measure of P815 cell killing. The P815 cells were incubated with mouse anti‐human CD3 antibody for 10 min in the incubator to facilitate cytotoxic activity by linking the CD3‐expressing T cells and the mouse Fc receptor‐expressing P815 cells. Next, the P815 and cytotoxic T cells were co‐cultured at a 1 : 5 ratio (T cell‐to‐P815 cell ratio) for 4 h. P815 cells without the addition of cytotoxic cells and P815 cells with the addition of 2% Triton X‐100 were also included as negative and positive controls, respectively. The cell suspension was then collected and spun down (5000 ***g***, 5 min), and the supernatant was harvested. Calcein release was measured in triplicates as absorbance using 495/515 excitation and emission filters on an Envision plate reader (PerkinElmer). The percentage killing was calculated in the sample relative to the positive control and corrected for effect in the negative control:

$$\%\text{Killing}=\frac{\left(\text{Absorbance in sample}-\text{Absorbance in negative control}\right)}{\left(\text{Absorbance in positive control}-\text{Absorbance in negative control}\right)}$$

## 3 Results

### 3.1 Roginolisib and idelalisib inhibit PI3K signaling and reduce the viability of CLL B cells

**Fig. 1:**
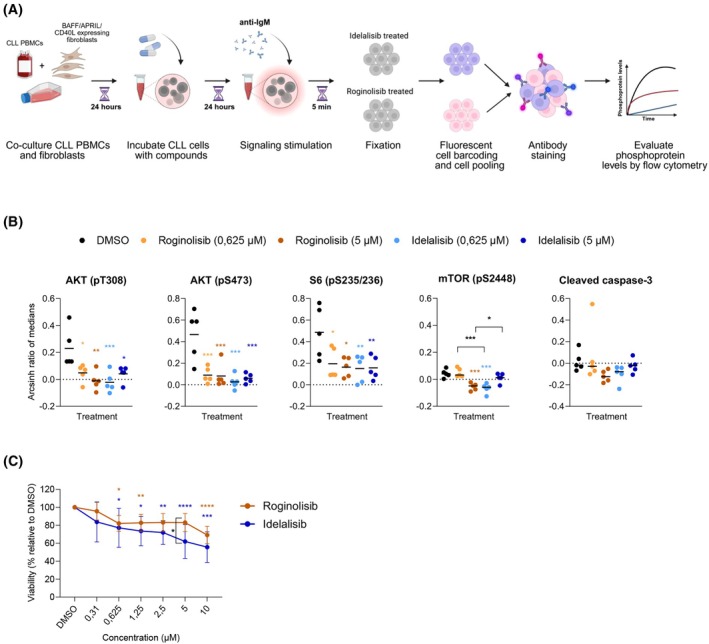
Roginolisib and idelalisib inhibit PI3K signaling and reduce viability of chronic lymphocytic leukemia (CLL) B cells. (A) Schematic overview of the phosphoflow workflow. Peripheral blood mononuclear cells (PBMCs) were isolated from CLL patients and co‐cultured 24 h with fibroblasts expressing BAFF, APRIL or CD40L to prevent spontaneous apoptosis of the CLL cells. Next, the CLL cells were separated from the fibroblasts and treated with DMSO (0.1%) or the indicated concentrations of idelalisib or roginolisib for another 24 h. B‐cell signaling was induced by anti‐IgM treatment and the cells were fixed after 5 min. The cell samples were barcoded using a combination of fluorescent dyes, so that the cells of each condition could later be separated based on their staining profile. The cells were then pooled together before being permeabilized and stained with antibodies against phosphospecific proteins and anti‐CD19, before being analyzed by flow cytometry. (B) CD19^+^ B cells were gated from the CLL PBMC population and levels of phospho‐epitopes in this population are expressed as arcsinh ratios after normalization of median intensity to that of unstimulated cells (no anti‐IgM), which was set to zero (*n* = 5). Statistical analyses were conducted by two‐way ANOVA analysis corrected for multiple comparisons: *P < 0.05, **P < 0.01, ***P < 0.001. Nonsignificant comparisons are not indicated. Gating strategy and representative staining intensity plots are shown in Fig. S1A,B. (C) CLL cells were isolated from CLL patients and co‐cultured for 24 h with fibroblasts expressing BAFF, APRIL and CD40L to prevent spontaneous apoptosis of the CLL cells. Next, the CLL cells were separated from the fibroblasts and treated with 0.1% DMSO, or the indicated concentrations of idelalisib or roginolisib for 72 h. Viability was assessed by luminescence (CellTitrerGlo). Data are represented as mean ± SD (*n* = 5). Statistical analyses were conducted by two‐way ANOVA analysis corrected for multiple comparisons: *P < 0.05, **P < 0.01, ***P < 0.001, ****P < 0.0001. Nonsignificant comparisons are not indicated.

To compare the effects of roginolisib and idelalisib, we treated isolated CLL PBMCs with the drugs for 24 h and evaluated their effects on PI3K‐related cell signaling (Fig. 1A). Patient characteristics are shown in Table S1. We found that both roginolisib and idelalisib inhibited PI3K signaling effectively, by reducing phosphorylation of key proteins downstream of PI3K (Fig. 1B, gating strategy and representative staining plots are shown in Fig. S1A,B). Roginolisib and idelalisib reduced AKT (pT308), AKT (pS473), and S6 ribosomal protein (pS235/236) phosphorylation to a similar degree at both concentrations tested, although the lowest concentration of roginolisib (0.625 μm) caused a less significant reduction (Fig. 1B). mTOR (pS2448) phosphorylation was only significantly reduced by the lowest concentration of idelalisib (0.625 μm) and the highest concentration of roginolisib (5 μm), which may indicate different effective concentration ranges for the two compounds. mTOR phosphorylation was also significantly different between roginolisib and idelalisib at both 0.625 μm (*P* = 0.0001) and 5 μm (*P* = 0.0297). We did not find that treatment with the compounds significantly affected cleavage of caspase‐3, involved in cell apoptosis (Fig. 1B).

Next, we performed cell viability assays and found that both idelalisib and roginolisib caused a modest but significant reduction in cell viability of up to 30–40% (Fig. 1C). Idelalisib caused a stronger reduction in viability compared with roginolisib, but the difference between the drugs was significant only at 5 μm. In summary, both drugs reduce PI3K signaling and viability in CLL cells, validating the on‐target function of these PI3K inhibitors in malignant B cells.

### 3.2 In contrast to idelalisib, roginolisib has a limited impact on the proliferation and function of CD8 ^+^ T cells

**Fig. 2:**
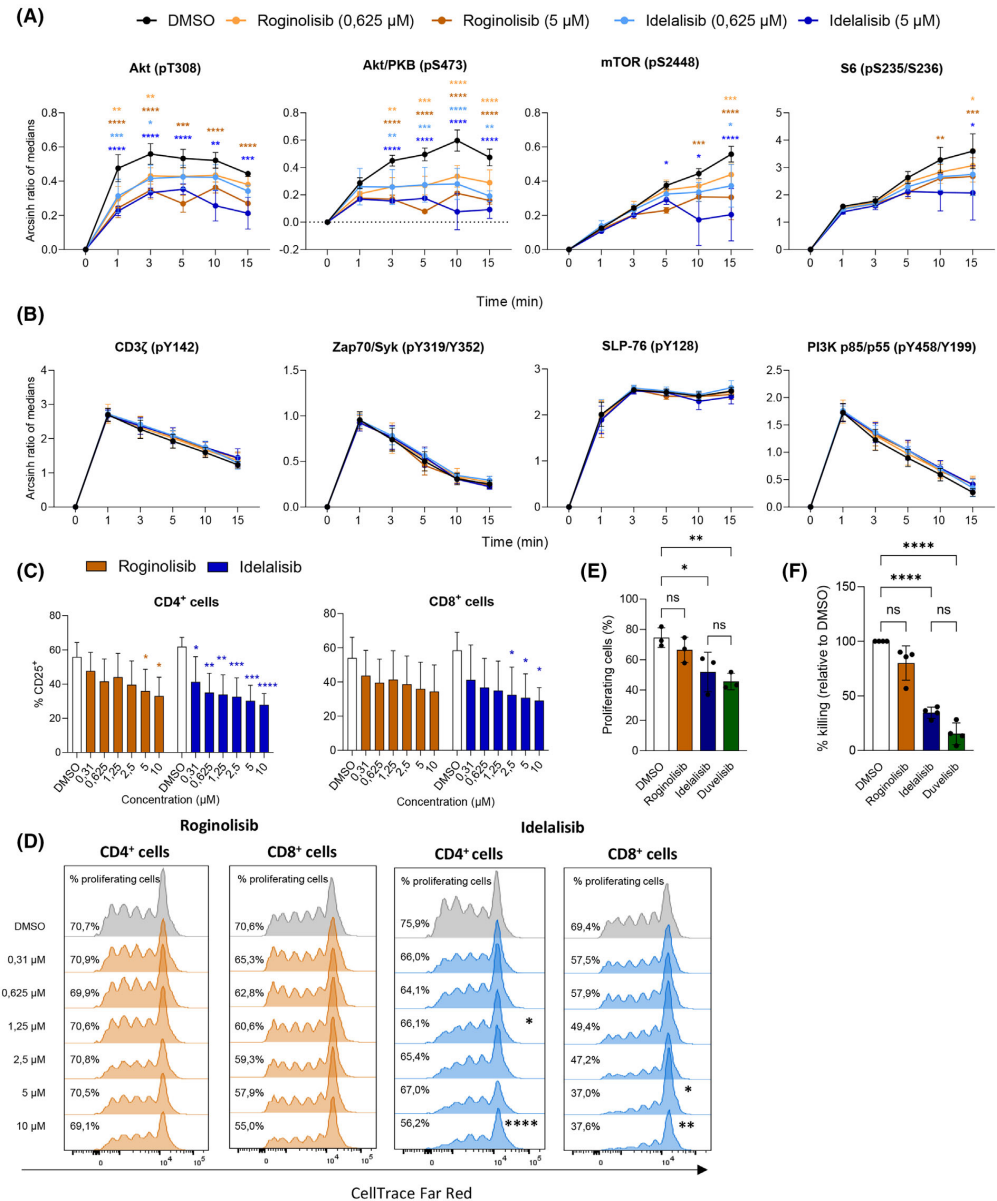
In contrast to idelalisib, roginolisib has limited impact on proliferation and function of CD8^+^ T. (A, B) CD3^+^ T cells were isolated from healthy donor buffy coats and treated with DMSO (0.1%) or the indicated concentrations of idelalisib or roginolisib for 30 min. Next, T‐cell receptor (TCR) signaling was initiated by anti‐CD3, anti‐CD28, and anti‐CD2 cross‐ligation. Cells were fixed at the indicated timepoints, before being fluorescently barcoded, permeabilized, and stained with phosphoprotein‐specific antibodies and anti‐CD4 and anti‐CD8. The cells were analyzed by flow cytometry. Graphs show mean ± SD (*n* = 3). Statistical analyses were conducted by two‐way ANOVA analysis corrected for multiple comparisons: *P < 0.05, **P < 0.01, ***P < 0.001, ****P < 0.0001. Nonsignificant comparisons are not indicated. Gating strategy and representative staining intensity plots are shown in Fig. S1C,D. (C) Healthy donor CD3^+^ T cells were treated with DMSO (0.1%) or the indicated concentrations of roginolisib or idelalisib for 48 h with anti‐CD3/2/28‐coated beads, before CD25 expression was evaluated by flow cytometry. Data are presented as mean ± SD (*n* = 5). Statistical analyses were conducted by two‐way ANOVA analysis corrected for multiple comparisons: *P < 0.05, **P < 0.01, ***P < 0.001, ****P < 0.0001. Nonsignificant comparisons are not indicated. (D) CD3^+^ T cells were stained with CellTrace CFSE dye to assess proliferation, and next stimulated with anti‐CD3/2/28‐coated and treated with DMSO (0.1%) or indicated concentrations of roginolisib or idelalisib for 5 days, before proliferation was assessed by flow cytometry. The percentage of proliferating cells is indicated for each condition. The figure shows representative flow cytometry plots from *n* = 3 donors. Aggregated data for roginolisib and idelalisib at 5 μm is shown in E. (E) Experiment was conducted as described in D, with duvelisib included. Data show aggregated results from 5 μm drug treatment (mean ± SD, *n* = 3). Statistical analyses were conducted by two‐way ANOVA analysis corrected for multiple comparisons. *P < 0.05, **P < 0.01, ns = nonsignificant. (F) CD8^+^ T cells were isolated from healthy donor buffy coats, stimulated with anti‐CD3/2/28‐coated beads and treated with DMSO (0.1%) or 5 μm of roginolisib, idelalisib, or duvelisib for 7 days. Next, the cells were co‐cultured with the murine mastocytoma cell line P815 for 4 h. The P815 cells were stained with calcein prior to co‐culture, and calcein release was used as a measure of P815 cell death and CD8+ T‐cell‐mediated killing (error bars indicate SD, *n* = 3). Statistical analyses were conducted by two‐way ANOVA analysis corrected for multiple comparisons: ****P < 0.0001. Nonsignificant comparisons are not indicated.

To investigate how roginolisib and idelalisib affect other cell subsets, we studied their effects on T‐cell receptor (TCR) signaling in human T cells. We found that the downstream targets of PI3K, particularly AKT, mTOR, and S6 ribosomal protein were significantly inhibited by both compounds (Fig. 2A, gating strategy and representative staining plots are shown in Fig. S1C,D), while proximal TCR‐signaling proteins, including CD3ζ, Zap70, SLP‐76, and the regulatory subunit of PI3K, remained unaffected (Fig. 2B, gating strategy and representative staining plots are shown in Fig. S1C,D). These findings support the notion of roginolisib specificity. The effects of roginolisib and idelalisib were concentration‐dependent and comparable (Fig. 2A). There were no prominent differences in the drug responses between CD4^+^ and CD8^+^ T cells (Fig. S2A,B).

PI3K activity is important for transmitting the TCR‐mediated activation signal. Thus, we evaluated how treatment with the compounds affected the expression of activation markers expressed on T cells. We found that roginolisib reduced CD25 expression significantly only in the CD4^+^ population, while idelalisib significantly reduced expression of CD25 on both CD4^+^ and CD8^+^ T cells (Fig. 2C). We also evaluated changes in other markers involved in stemness and exhaustion (PD‐1, TCF‐1, and TOX), and only idelalisib reduced TCF‐1 expression significantly in CD4^+^ cells (Fig. S2C). Roginolisib only marginally inhibited the proliferation of CD8^+^ T cells, and not of CD4^+^ cells (Fig. 2D,E). Idelalisib, however, inhibited proliferation of both CD4^+^ and CD8+ T cells, most prominently CD8^+^ T cells (Fig. 2D,E). Since CD8^+^ T‐cell proliferation seemed to be more strongly inhibited by both compounds, we further investigated the functional effects of these compounds on cytotoxic T cells by conducting a cytotoxic T‐cell killing assay. Here, CD8^+^ T cells were preactivated by anti‐CD3/28‐coated beads in the presence or absence of the drugs before being co‐cultured with target murine P815 tumor cells. We found that treatment with idelalisib significantly reduced the cytotoxic killing capacity, while roginolisib‐treated cells did not significantly alter the killing capacity compared with the control (Fig. 2F). To investigate whether the stronger suppression of cytotoxic functions by idelalisib could be due to it also affecting other PI3K isozymes, we evaluated effect on proliferation and conducted the killing assay with the dual PI3Kδ/γ inhibitor duvelisib. We found that duvelisib suppressed cytotoxic functions and proliferation in similar manners as idelalisib (Fig. 2E,F), suggesting that the detrimental effect of idelalisib on T‐cell killing could be due to its additional effects on PI3Kγ. This off‐target effect was not observed with roginolisib.

In summary, roginolisib and idelalisib inhibit PI3K T‐cell signaling and activation in a comparable manner. Idelalisib most prominently inhibited CD8^+^ and CD4^+^ T‐cell proliferation, and high concentrations of idelalisib strongly reduced CD8^+^ T‐cell killing capacities. Roginolisib did not significantly reduce T‐cell proliferation or killing capacity.

### 3.3 Roginolisib and idelalisib modestly affects FoxP3 expression and Treg suppressive functions

**Fig. 3:**
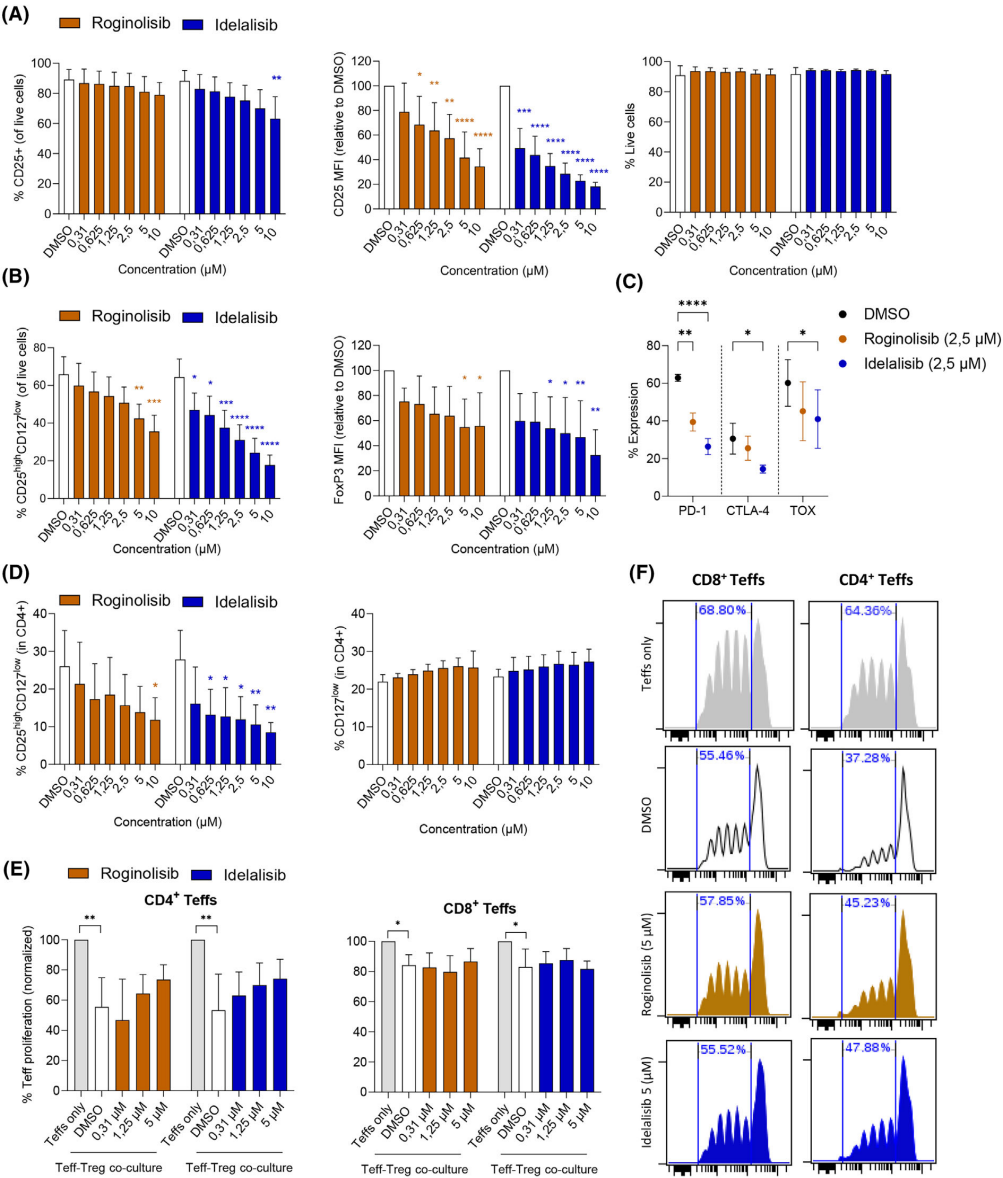
Idelalisib and roginolisib modestly regulate the expression of Treg activation markers and function. (A) Regulatory T cells (Tregs) were isolated from healthy donors, stimulated with anti‐CD3/2/28‐coated beads and treated with DMSO (0.1%) or indicated concentrations of roginolisib or idelalisib for 48 h. The percentage of CD25^+^ cells (left panel), CD25 median fluorescent intensity (MFI) (middle panel) and viability (by Fixable Viability Stain, right panel) were assessed by flow cytometry. Data are represented as mean ± SD (*n* = 4). Statistical analyses were conducted by two‐way ANOVA analysis corrected for multiple comparisons: *P < 0.05, **P < 0.01, ***P < 0.001, ****P < 0.0001. Nonsignificant comparisons are not indicated. Representative flow gating is shown in Fig. S3. (B) Tregs were isolated and treated as described in (A), and the percentage of CD25^high^CD127^low^ (left panel) and FoxP3 MFI (right panel) were assessed by flow cytometry. Data are represented as mean ± SD (*n* = 4). Statistical analyses were conducted by two‐way ANOVA analysis corrected for multiple comparisons: *P < 0.05, **P < 0.01, ***P < 0.001, ****P < 0.0001. Nonsignificant comparisons are not indicated. Representative flow gating is shown in Fig. S3. (C) Tregs were isolated and treated as described in (A) and stained with antibodies against the indicated T‐cell activation and stemness markers. Data are represented as mean ± SD (*n* = 3). Statistical analyses were conducted by two‐way ANOVA analysis corrected for multiple comparisons: *P < 0.05, **P < 0.01, ****P < 0.0001. Nonsignificant comparisons are not indicated. (D) CD3^+^ T cells were isolated from healthy donors and treated with DMSO (0.1%) or indicated concentrations of roginolisib or idelalisib and anti‐CD3/2/28‐coated beads for 48 h. Data are represented as mean ± SD (*n* = 3). The percentage of CD25^high^CD127^low^ cells was assessed by flow cytometry. Statistical analyses were conducted by two‐way ANOVA analysis corrected for multiple comparisons: *P < 0.05, **P < 0.01. Nonsignificant comparisons are not indicated. (E) Effects of roginolisib and idelalisib on Treg‐mediated suppression. Isolated Tregs were stimulated with anti‐CD3/2/28‐coated beads and treated with DMSO (0.1%) or indicated concentrations of roginolisib or idelalisib for 48 h. Next, the Tregs were washed, counted and co‐cultured with isolated CD4^+^ or CD8^+^ T effector cells (Teffs) at a ratio of 1 : 5 for 4 days. The T effector cells were stained with CFSE CellTrace dye prior to co‐culture to enable measurement of T effector cell proliferation. Treg‐mediated suppression was measured indirectly as T effector cell proliferation by flow cytometry. T effector cell proliferation was normalized to proliferation without Tregs (Teffs only). Data are represented as mean ± SD (*n* = 4). Statistical analyses were conducted by two‐way ANOVA analysis corrected for multiple comparisons: *P < 0.05, **P < 0.01. Nonsignificant comparisons are not indicated. (F) Representative flow cytometry plots of data shown in (E). Representative flow gating is shown in Fig. S4.

Since both roginolisib and idelalisib inhibited expression of CD25, a protein highly expressed by Tregs, we investigated how these compounds affect Treg function. We isolated Tregs from CD3^+^ T cells from healthy donor blood and treated the Tregs with either compound. We confirmed that CD25 expression levels were reduced by the treatment (Fig. 3A, left and middle panel), and that this reduction was likely not due to toxicity, as none of the compounds reduced the viability of the isolated Tregs (Fig. 3A, right panel). Next, we evaluated the expression of other Treg markers or discriminators in the isolated Treg population. The levels of CD25^high^CD127^low^ Tregs were also reduced, most significantly by idelalisib at all concentrations tested (Fig. 3B, left panel). FoxP3 expression levels were also reduced most significantly by idelalisib at a concentration of 1.25 μm and above (Fig. 3B, right panel). We next studied expression of other relevant activation markers, including PD‐1, CTLA‐4, and TOX. Although treatment with either compound reduced expression of these markers, only PD‐1 was significantly reduced by both compounds, while only idelalisib reduced CTLA‐4 and TOX expression significantly (Fig. 3C). The reduction of the CD25^high^CD127^low^ population was comparable in the total T‐cell population (Fig. 3D, left panel). These cells were significantly reduced by idelalisib across all concentrations above 0.625 μm, while only significantly at 10 μm roginolisib (Fig. 3D, left panel). The CD127^low^ population was not reduced by any of the compounds (Fig. 3D, right panel), so the reduction in the CD25^high^CD127^low^ cells in the total T‐cell population may be mostly due to the reduction in CD25 expression.

To evaluate whether Treg suppressive function was affected by the drugs, we conducted a Treg suppression assay. Isolated Tregs were activated by anti‐CD3/2/28‐coated beads and treated with the drugs for 48 h before being co‐cultured with isolated CD4^+^ or CD8^+^ T effector cells for 96 h. Treatment with either drug tended to restore proliferation of CD4^+^ T cells, whereas proliferation of CD8^+^ T cells was not restored by any of the treatments (Fig. 3E,F). These findings are in line with the modest inhibitory effects both drugs had on FoxP3 levels (Fig. 3B, right panel), as FoxP3 is a main regulator of Treg suppressive functions.

### 3.4 Idelalisib, and not roginolisib, promotes conventional T‐cell subsets

**Fig. 4:**
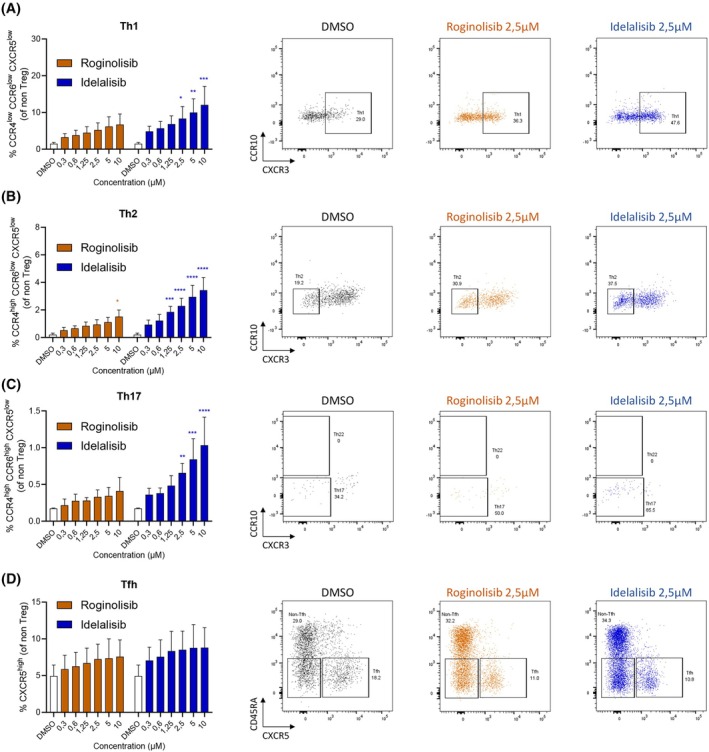
Idelalisib, but not roginolisib, upregulated conventional T cells subsets. CD4^+^ T cells were isolated from the blood of healthy donors, activated with anti‐CD3/28 and treated for 48 h with idelalisib, roginolisib, or DMSO. (A) The percentage of CCR4^low^CCR6^low^CXCR5^low^ Th1 cells out of non‐Tregs (CD4^+^CD25^low^) was assessed by flow cytometry. Data are represented as mean ± SD (*n* = 3). Statistical analyses were conducted by two‐way ANOVA analysis corrected for multiple comparisons: *P < 0.05, **P < 0.01, ***P < 0.001. Nonsignificant comparisons are not indicated. (B) The percentage of CCR4^high^CCR6^low^CXCR5^low^ Th2 cells out of non‐Tregs was assessed by flow cytometry. Data are represented as mean ± SD (*n* = 3). Statistical analyses were conducted by two‐way ANOVA analysis corrected for multiple comparisons: *P < 0.05, ***P < 0.001, ****P < 0.0001. Nonsignificant comparisons are not indicated. (C) The percentage of CCR4^high^CCR6^high^CXCR5^low^ Th17 cells out of non‐Tregs was assessed by flow cytometry. Data are represented as mean ± SD (*n* = 3). Statistical analyses were conducted by two‐way ANOVA analysis corrected for multiple comparisons: **P < 0.01, ***P < 0.001, ****P < 0.0001. Nonsignificant comparisons are not indicated. (D) The percentage of CXCR5^high^ follicular helper T cells was assessed by flow cytometry. Data are represented as mean ± SD (*n* = 3). Statistical analyses were conducted by two‐way ANOVA analysis corrected for multiple comparisons. Nonsignificant comparisons are not indicated. Representative flow gating is shown in Fig. S5.

In addition to regulatory T cells, we also investigated the impact of PI3Kδ inhibition on conventional CD4^+^ T cell subsets. CD4^+^ T cells were isolated from the peripheral blood of healthy donors and treated with either idelalisib or roginolisib at indicated concentrations. Flow cytometry analysis was performed to quantify distinct T helper populations based on the expression of specific chemokine receptors. Idelalisib induced a concentration‐dependent increase in the percentage of CCR4^low^CCR6^low^CXCR5^low^ Th1 cells, whereas roginolisib had no significant effect (Fig. 4A). A similar pattern was observed for CCR4^high^CCR6^low^CXCR5^low^ Th2 cells, where idelalisib concentrations equal to or above 1.25 μm led to an increase in cell percentage, while a significant effect from roginolisib was only observed at the highest concentration of 10 μm (Fig. 4B). Furthermore, idelalisib at 2.5 μm and above promoted expansion of CCR4^high^CCR6^high^CXCR5^low^ Th17 cells, while roginolisib again had no impact (Fig. 4C). Finally, despite a slight upward trend, neither drug significantly affected the CXCR5^high^ follicular helper T‐cell subset (Fig. 4D).

## 4 Discussion

Clinical use of PI3Ki has shown promise in treating some types of cancer, but at the expense of serious immune‐related adverse effects. This has led to black box warnings or retractions of some drugs from the market [7, 13]. Thus, there is interest in developing novel and effective PI3Ki that lack these harmful side effects. Here, we evaluated the effects of the novel PI3K inhibitor roginolisib (IOA‐244) compared with those of idelalisib. We found that both drugs specifically inhibited PI3K‐related signaling, verifying their on‐target effects. Both drugs also comparably inhibited CLL B‐cell viability, further demonstrating the efficacy of roginolisib. Idelalisib and roginolisib were also found to specifically inhibit PI3K‐related signaling in T cells. Importantly, roginolisib, in contrast to idelalisib, only mildly affected T‐cell proliferation and effector function. The same trend was found for Tregs, where idelalisib inhibited Treg activation more strongly compared with roginolisib. Idelalisib also more potently promoted CD4^+^ Th1, Th2, and Th17 cell subsets.

These findings are also in line with previous studies on roginolisib. Roginolisib was initially proposed as potential therapeutic for autoimmune disease [14], but later also showed to be beneficial in a cancer setting, where the drug both induces cancer cell apoptosis and enhances tumor immunity [15, 16]. One of these studies found that idelalisib and roginolisib inhibited Treg proliferation, while CD4^+^ and CD8^+^ T effector cell proliferation was only marginally reduced [15]. Here, we found that both drugs tended to inhibit T effector cell proliferation, but only idelalisib reduced proliferation significantly (Fig. 2D). These conflicting findings could be explained by the different concentration ranges employed in the different studies; in [15] the authors used lower concentrations of both drugs (0.001–1 μm), than what we used (0.31–10 μm). The concentrations used in this study are close to the maximum plasma concentration (*C*_max_) observed in human patients, which range from 2.2 to 7 μm for idelalisib and reach approximately 10 μm for roginolisib [8, 17]. The minimum concentrations (*C*_min_) for idelalisib and roginolisib were 0.2–1‐7 μm and 4.8 μm, respectively.

Some of the adverse effects from PI3Ki are believed to be due to their inhibitory effects on Tregs. Tregs regulate activity and function of CD4^+^ and CD8^+^ Teffs, and Treg inhibition could thus result in a pool of overactive Teffs. Consistent with our findings, idelalisib has also been shown to induce dysfunctional T‐cell subsets in *ex vivo* treatment of PBMCs isolated from both healthy donors and CLL patients [18]. Indeed, many of the adverse effects reported following administration of PI3Ki are likely connected to dysregulated Teffs [19]. Studies have also found a denser T‐cell infiltrate, accompanied by decreased Treg infiltration in affected tissues [19, 20, 21]. Our group and others have also previously shown inhibitory effects of PI3Ki on Treg suppressive functions [22, 23, 24]. However, Treg‐specific inhibition can also be beneficial in cancerous tumors characterized by a high Treg‐infiltrate. Here, the Tregs inhibited Teffs in killing the cancer cells, so specific targeting could, in this case, enhance tumor immunity [25].

Previous clinical studies have reported an enrichment of Th17 cells in CLL patients who developed immune‐related adverse events following idelalisib treatment [26]. Here, we show that idelalisib, but not roginolisib, selectively promotes the differentiation of CD4^+^ T cells into Th1, Th2, and Th17 subsets. The absence of Th17 expansion in response to roginolisib may reflect a more favorable safety profile, potentially associated with a lower risk of Th17‐driven toxicity.

Interestingly, the key differences between idelalisib and roginolisib observed in this study primarily emerged from immune cell‐based functional assays, rather than from biochemical analyses or their efficacy in lymphoma cell cytotoxicity. This suggests that some of the immunological effects cannot be solely attributed to differences in potency. Notably, unlike idelalisib, roginolisib is a non‐ATP competitive inhibitor of PI3Kδ and operates via a distinct binding mechanism [27]. Whether this alternative mode of inhibition underlies some of the observed immunophenotypic differences remains to be elucidated in future studies.

Roginolisib appears to exhibit higher specificity toward PI3Kδ when compared to idelalisib at clinically relevant concentrations [15]. This difference in off‐target activity may explain the less detrimental impact of roginolisib on T‐cell functions, as it targets fewer additional PI3K subunits at concentrations investigated. Other studies indicate that inhibiting PI3K during preparation of adoptively transferred T cells enhances their functionality [28, 29, 30, 31, 32, 33]. Notably, one study reported that the dual PI3Kδ/γ inhibitor duvelisib diminished the antitumor potential of CAR T cells, while CAR T cells treated with either a PI3Kδ inhibitor or a PI3Kγ inhibitor alone, rather displayed improved antitumor activity [28]. We have also found that treatment with duvelisib severely compromised T‐cell cytotoxic functions compared with roginolisib (Fig. 2E). Therefore, developing highly selective PI3K inhibitors targeting individual subunits could significantly enhance T‐cell functions. Overall, roginolisib presents a promising alternative to earlier‐generation PI3Kδ inhibitors, and the results of ongoing clinical trials will be crucial to determine whether its potentially improved safety profile will support broader clinical application.

## 5 Conclusions

The promise of PI3Ki as cancer therapeutics has been hampered by severe immune‐related adverse effects. Thus, developing novel PI3Ki that lack these adverse effects is of great interest. Here, we compared the effects of the investigational PI3K inhibitor roginolisib to that of the approved PI3K inhibitor idelalisib. We found that roginolisib and idelalisib inhibit CLL cells and Treg suppressive functions to similar extents, but roginolisib affects cytotoxic T‐cell function and promotion of pro‐inflammatory T helper subsets to a much lesser extent than idelalisib. Thus, roginolisib poses as an attractive alternative to current PI3Ki.

## Conflict of interest

S.S.S. has received consulting fees from AstraZeneca, BeiGene, and Janssen, and has received research support from BeiGene and TG Therapeutics. A.B. and G.D.C. are employees at iOnctura SA. M.W. and D.M. received funding from iOnctura SA.

## Author contributions

K.T. and G.D.C. designed the study. E.S., D.M., M.W., and A.B. performed the experiments and analyzed the data. E.S., A.B., and G.D.C. wrote the article. L.vdV reviewed the manuscript. K.T., S.S.S., L.vdV, and G.D.C. supervised the study. All authors read and commented on the manuscript and approved the final version.

## Supporting information


**Fig. S1.** Relevant gating strategy and representative staining.

**Fig. S2.** Effect on cell signaling and expression of T‐cell activation markers in CD4+ or CD8+ T cells.

**Fig. S3.** Gating strategy for results shown in Fig. 3A,B.

**Fig. S4.** Gating strategy for results shown in Fig. 3E,F.

**Fig. S5.** Gating strategy for results shown in Fig. 4A–D.

**Table S1.** CLL patient characteristics.

## Data Availability

The data that support the findings of this study are available from the corresponding author upon reasonable request.
