# Supplementary figures and images for: The PI3Kδ inhibitor roginolisib (IOA‐244) preserves T‐cell function and activity
Source: Mol Oncol. 2026 Jan 22;20(6):1612–25. doi: 10.1002/1878-0261.70203 (PMC13238606; doi:10.1002/1878-0261.70203)

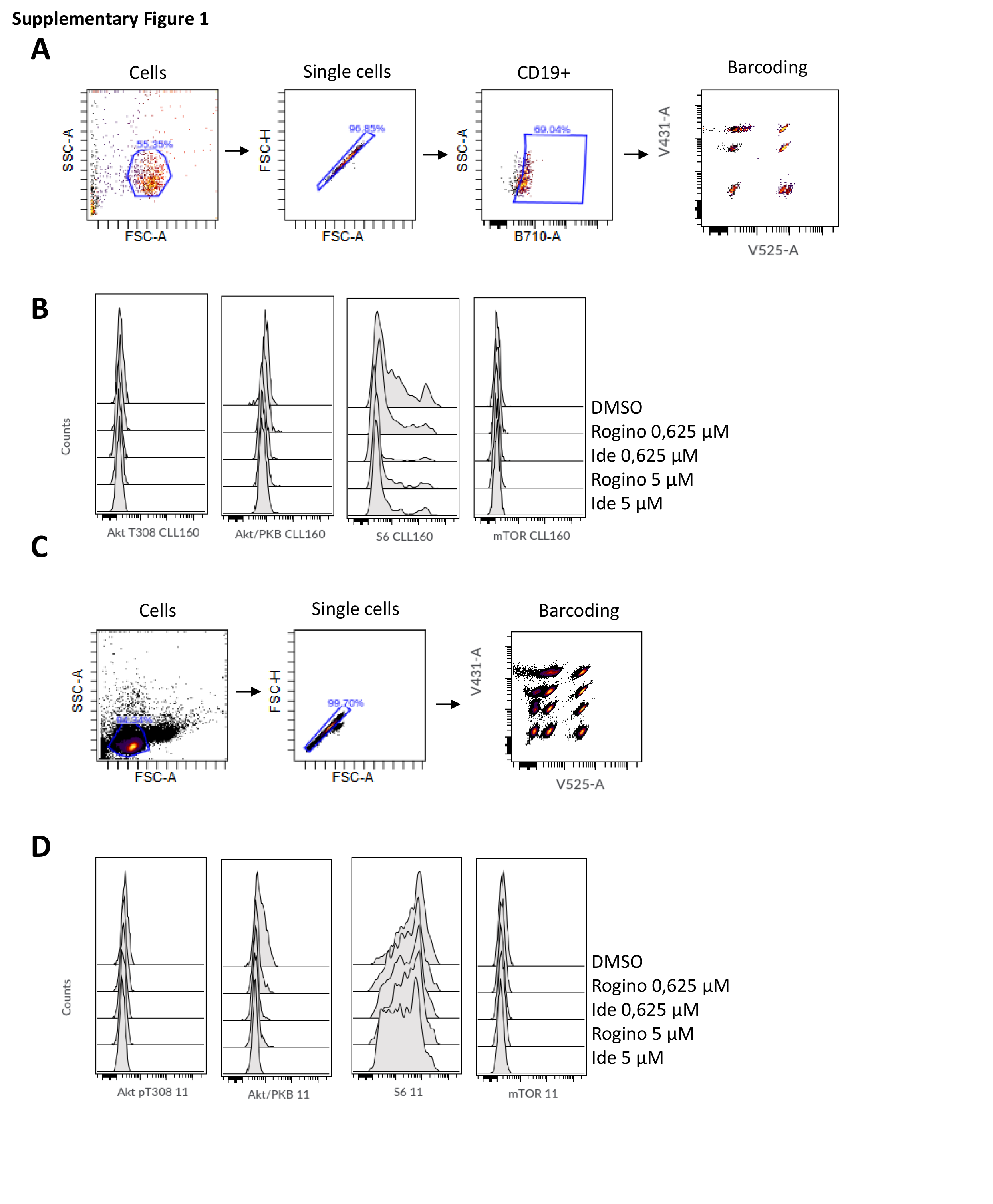

Supplement: Supplementary file 1 — Fig. S1. Relevant gating strategy and representative staining. [file MOL2-20-1612-s003.tif]

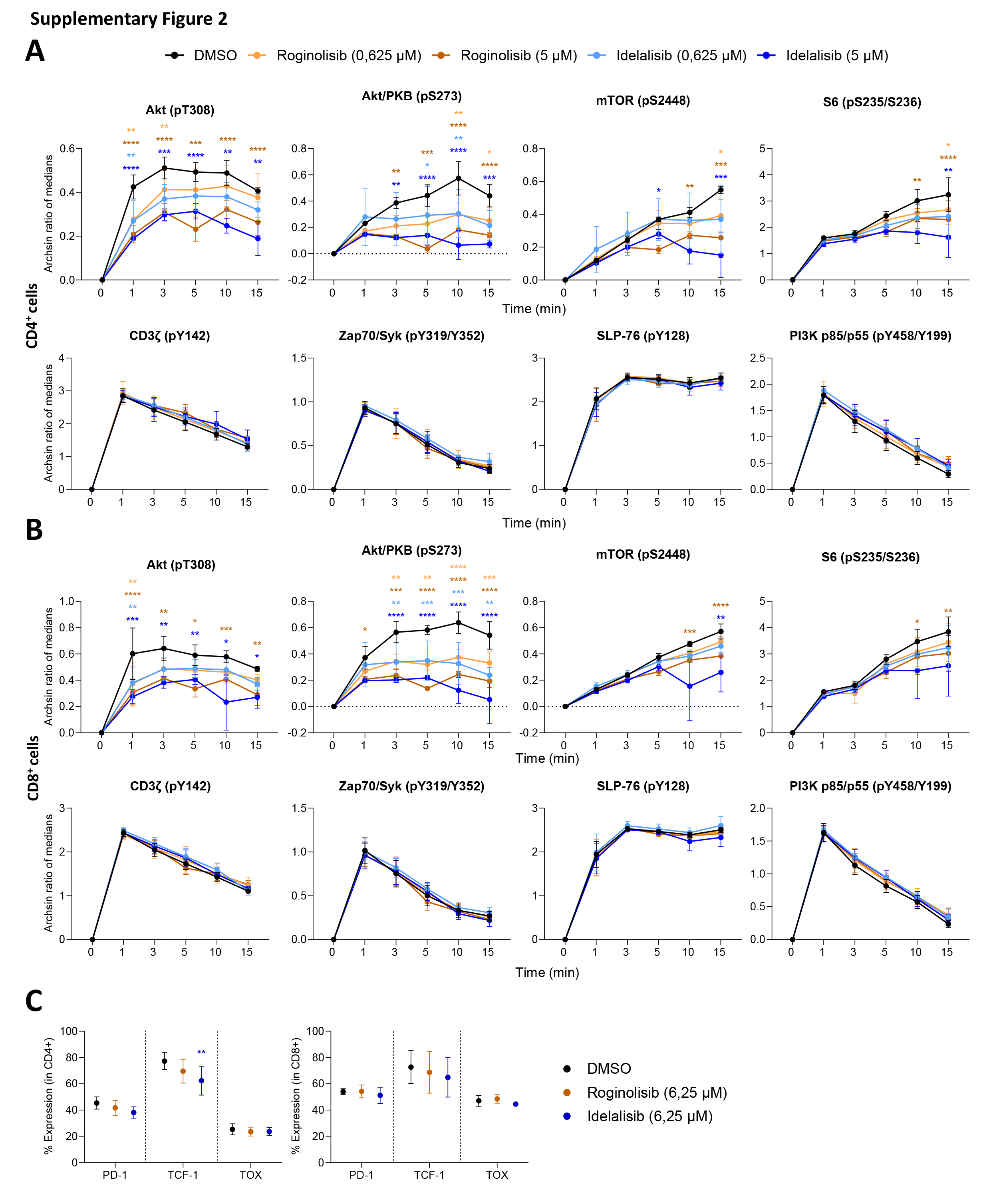

Supplement: Supplementary file 2 — Fig. S2. Effect on cell signaling and expression of T‐cell activation markers in CD4+ or CD8+ T cells. [file MOL2-20-1612-s004.tif]

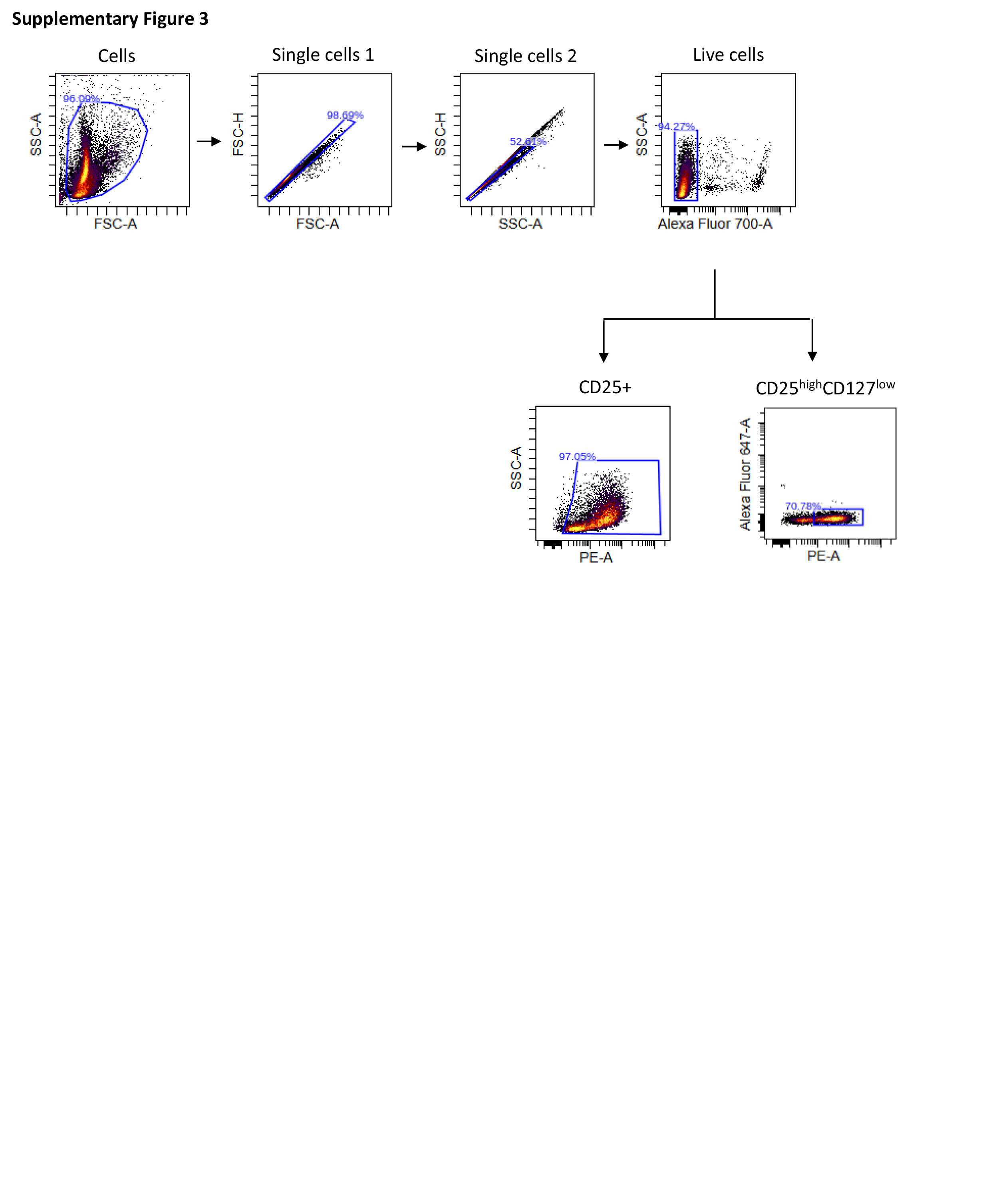

Supplement: Supplementary file 3 — Fig. S3. Gating strategy for results shown in Fig. 3A,B. [file MOL2-20-1612-s006.tif]

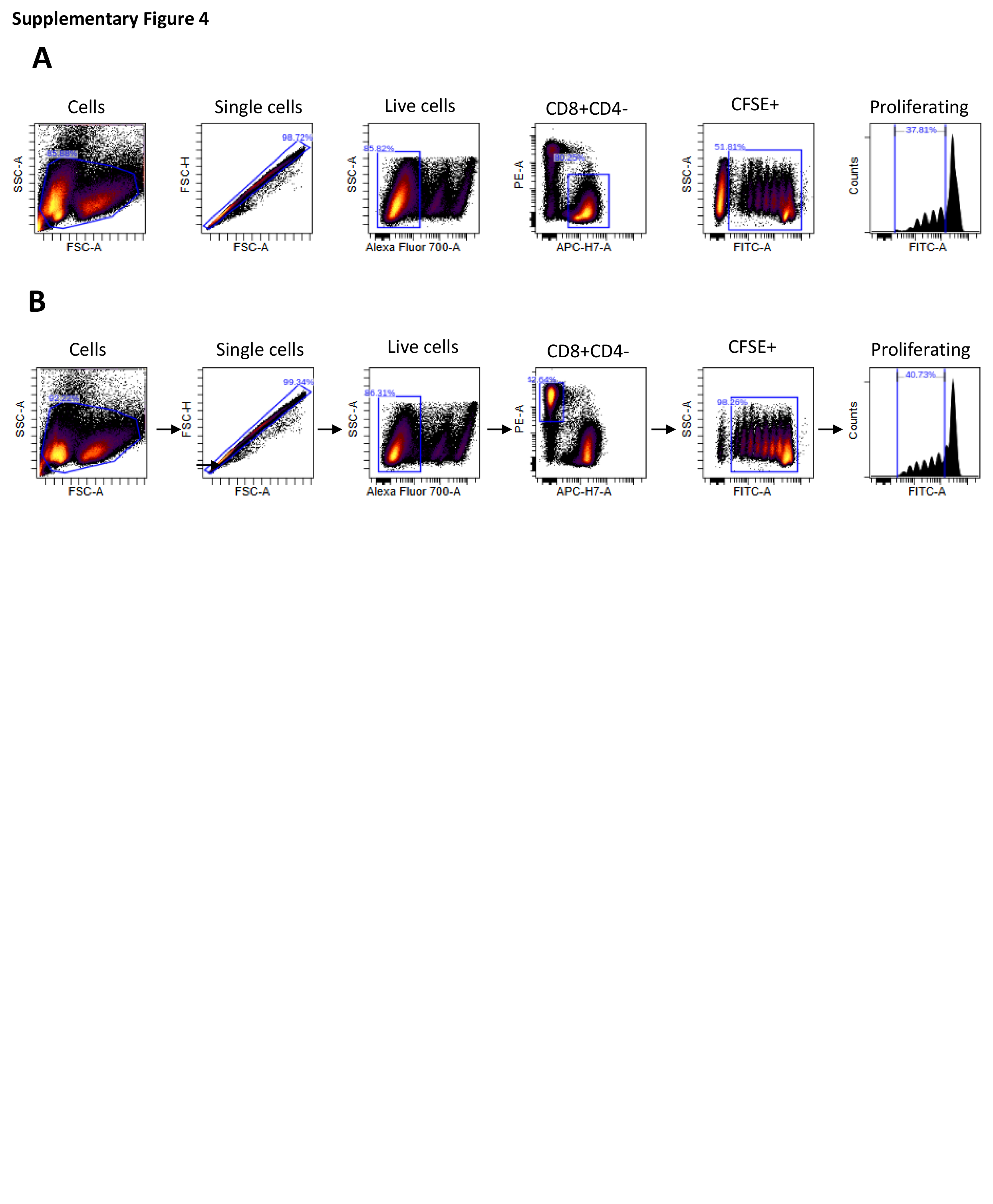

Supplement: Supplementary file 4 — Fig. S4. Gating strategy for results shown in Fig. 3E,F. [file MOL2-20-1612-s001.tif]

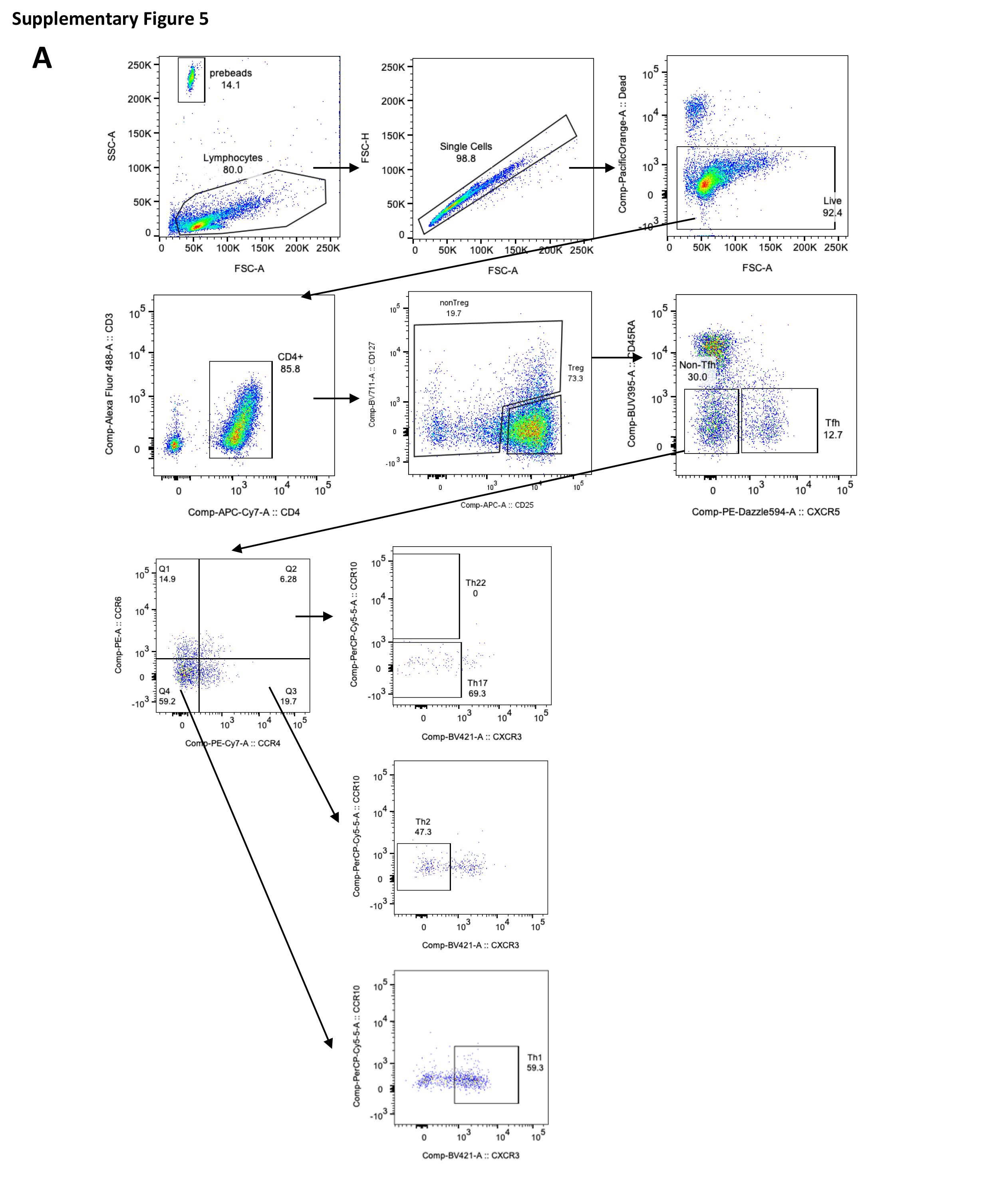

Supplement: Supplementary file 5 — Fig. S5. Gating strategy for results shown in Fig. 4A–D. [file MOL2-20-1612-s005.tif]

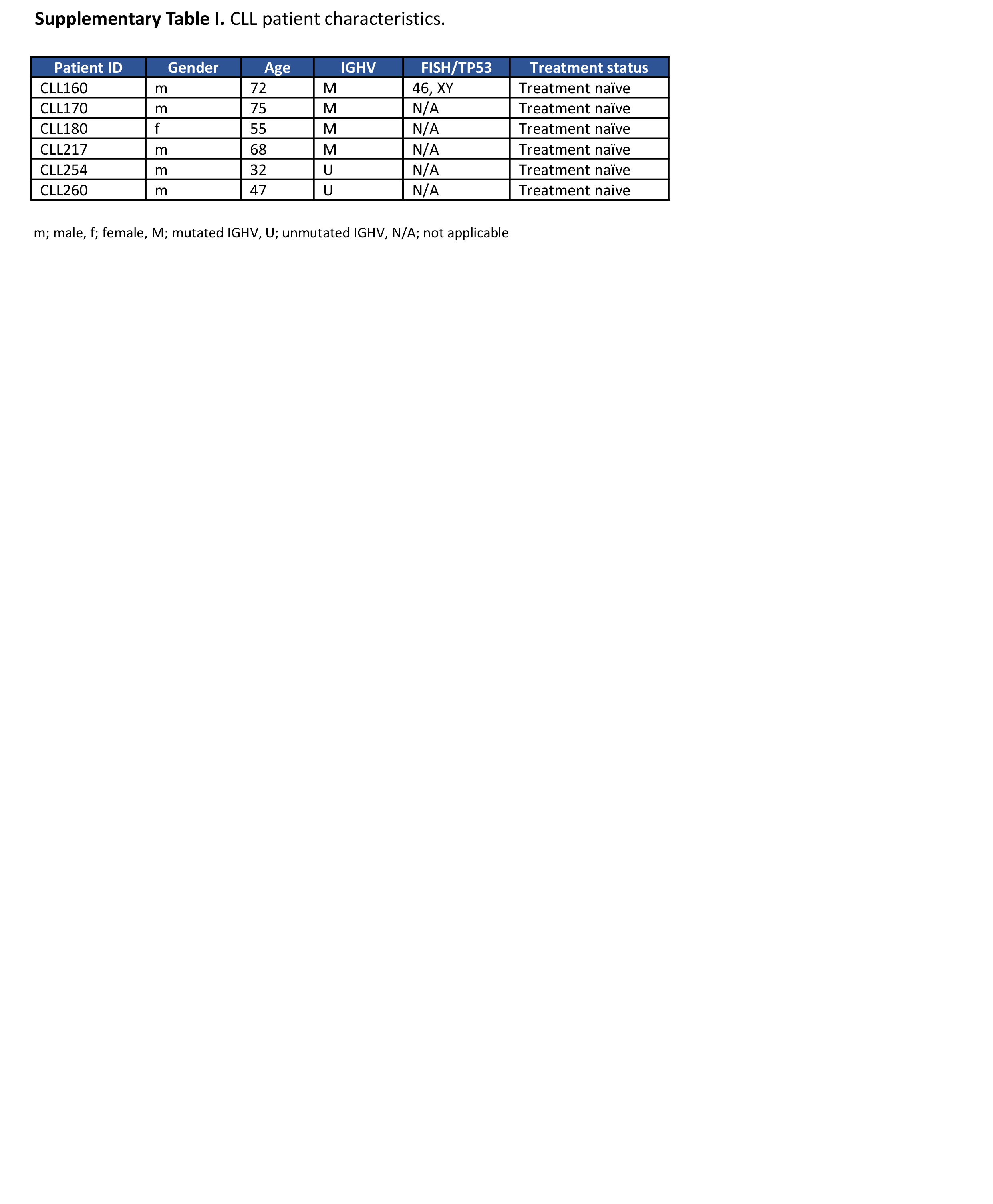

Supplement: Supplementary file 6 — Table S1. CLL patient characteristics. [file MOL2-20-1612-s002.tif]
